# Dependence on Dectin-1 Varies With Multiple *Candida* Species

**DOI:** 10.3389/fmicb.2019.01800

**Published:** 2019-08-06

**Authors:** Aiysha Thompson, James S. Griffiths, Louise Walker, Diogo M. da Fonseca, Keunsook K. Lee, Philip R. Taylor, Neil A. R. Gow, Selinda J. Orr

**Affiliations:** ^1^Division of Infection and Immunity and Systems Immunity Research Institute, School of Medicine, Cardiff University, Cardiff, United Kingdom; ^2^UK Dementia Research Institute, Cardiff University, Cardiff, United Kingdom; ^3^Medical Research Council Centre for Medical Mycology, Aberdeen Fungal Group, University of Aberdeen, Aberdeen, United Kingdom; ^4^School of Biosciences, University of Exeter, Exeter, United Kingdom

**Keywords:** Dectin-1, *Candida* spp., macrophages, dendritic cells, T cells

## Abstract

Four *Candida* spp. (*albicans*, *glabrata*, *tropicalis*, *parapsilosis*) cause >95% of invasive *Candida* infections. *C. albicans* elicits immune responses via pathogen recognition receptors including C-type lectin-like receptors (CLRs). The CLR, Dectin-1 is important for host immunity to *C. albicans* and *C. glabrata*, however, whether Dectin-1 is important for host defense against *C. tropicalis* or *C. parapsilosis* is unknown. Therefore, we compared the involvement of Dectin-1 in response to these four diverse *Candida* spp. We found that Dectin-1 mediates innate cytokine responses to these *Candida* spp. in a species- and cell-dependent manner. Dectin-1 KO mice succumbed to infection with highly virulent *C. albicans* while they mostly survived infection with less virulent *Candida* spp. However, Dectin-1 KO mice displayed increased fungal burden following infection with each *Candida* spp. Additionally, T cells from Dectin-1 KO mice displayed enhanced effector functions likely due to the inability of Dectin-1 KO mice to clear the infections. Together, these data indicate that Dectin-1 is important for host defense to multiple *Candida* spp., although the specific roles for Dectin-1 varies with different *Candida* spp.

## Introduction

*Candida* spp. cause ∼400,000 life-threatening invasive infections per year with mortality rates of 46–75% (Brown et al., 2012). They are the fourth most common pathogens causing hospital acquired infections (HAI) (Hidron et al., 2008; Alangaden, 2011), and they are the most common fungal pathogens causing life-threatening HAIs particularly in immunosuppressed patients (Alangaden, 2011). *Candida albicans* is the most frequent cause, however, other *Candida* spp. including *C. glabrata*, *C. tropicalis*, and *C. parapsilosis* are also frequently isolated from patients with invasive candidiasis. In addition, smaller numbers of cases of *C. krusei*, *C. guilliermondii*, *C. kefyr*, *C. dubliniensis*, and *C. zeylanoides* infection have been identified (Alangaden, 2011; Karacaer et al., 2014). An emerging multidrug resistant *Candida* spp., *C. auris* is now appearing in hospitals across the world and will likely join the top four *Candida* spp. (*C. albicans*, *C. glabrata*, *C. tropicalis*, and *C. parapsilosis*) as a major cause of fungal HAIs in the next few years (Chowdhary et al., 2017).

*Candida* spp. differ in their biology and virulence. *C. albicans* is polymorphic as it can grow in yeast, pseudohyphal and hyphal forms, however, *C. glabrata* is monomorphic as it can only grow as blastoconidia (yeast). *C. parapsilosis* does not form true hyphae but it can grow in yeast form and it can produce psuedohyphae, whereas *C. tropicalis* can grow in yeast form, psuedohyphal form and in some reports it has been shown to form hyphae (Fidel et al., 1999; Trofa et al., 2008; Gow et al., 2011; Silva et al., 2012). Multiple studies have linked the formation of *C. albicans* hyphae with enhanced tissue invasion, damage and virulence (Lo et al., 1997; Gow et al., 2011). In agreement with this, comparison studies in mice have found that *C. albicans* is more pathogenic than any of the other *Candida* spp. tested (Anaissie et al., 1993; Brieland et al., 2001; Arendrup et al., 2002). Based on *in vivo* mortality rates and histological changes, Arendrup et al. divided 8 *Candida* spp. into three groups with decreasing virulence: (1) *C. albicans, C. tropicalis*, (2) *C. glabrata, C. kefyr, C. lusitaniae* and (3) *C. parapsilosis, C. krusie* and *C. guilliermondii* (Arendrup et al., 2002). According to this study, the top four *Candida* spp. (*C. albicans*, *C. glabrata*, *C. tropicalis*, and *C. parapsilosis*) that cause life-threatening HAIs in patients actually display highly different levels of pathogenicity, however, the immune responses induced by these different species have not been comprehensively compared.

The innate immune system is the front line of defense against pathogens including *C. albicans*. Pathogen associated molecular patterns such as mannans and ß1,3-glucans found in the cell wall of *C. albicans* are recognized by pathogen recognition receptors (PRRs) including C-type lectin-like receptors (CLRs) and Toll-like receptors (TLRs) (Patin et al., 2018). The CLR, Dectin-1, binds ß1,3-glucans in fungal cell walls. Dectin-1 is important for mediating/regulating immune responses to *C. albicans* and *C. glabrata* including phagocytosis, cytokine/chemokine production, respiratory burst, inflammatory cell recruitment, neutrophil extracellular traps and T cell responses (Taylor et al., 2007; Branzk et al., 2014; Chen et al., 2017). While Dectin-1 has been shown in many studies to be important for controlling systemic *C. albicans* infections, the requirement for Dectin-1 is actually fungal strain specific due to differences in chitin levels in their cell walls (Saijo et al., 2007; Marakalala et al., 2013). Dectin-1 KO mice infected with *C. albicans* displayed increased fungal burdens in the kidneys and gastrointestinal tract and the mice succumbed to the infection when infected with *C. albicans* strains that require Dectin-1 for clearance (Taylor et al., 2007; Marakalala et al., 2013). *C. glabrata*-infected Dectin-1 KO mice displayed increased fungal burdens in the kidneys and livers, however, they did not succumb to the infection (Chen et al., 2017). While *C. tropicalis* has been shown to induce Dectin-1 expression (Duan et al., 2018) and inhibition of Dectin-1 in peripheral blood mononuclear cells resulted in reduced *C. parapsilosis*-induced cytokine production (Toth et al., 2013), the role of Dectin-1 *in vivo* in response to these *Candida* spp. is unknown.

In the present study, we examined the role of Dectin-1 in response to the four main *Candida* spp. (*C. albicans*, *C. glabrata*, *C. tropicalis* and *C. parapsilosis*) responsible for life-threatening HAIs. We found that Dectin-1 mediates innate cytokine responses from macrophages and dendritic cells to these four *Candida* species in a species and cell dependent manner. We showed that Dectin-1 was required to help clear systemic infections with all four spp. In agreement with previous studies, we found that *C. albicans* was the most pathogenic strain and Dectin-1 KO mice succumbed to infection with *C. albicans* while they mostly survived infection with the other less pathogenic *Candida* spp. In addition, we found that Dectin-1 modulates some anti-fungal T cell responses. Our study shows that Dectin-1 plays an important role in host defense to multiple *Candida* spp., however, the requirement for Dectin-1 for specific immune responses varies with different *Candida* spp.

## Materials and Methods

### Mice

Dectin-1 KO (*Clec7a*^–/–^) (Taylor et al., 2007) and age and gender matched control C57BL/6 mice were maintained and handled according to institutional and UK Home Office regulations.

### Ethics Statement

All procedures were approved by the Cardiff University’s Animal Welfare and Ethical Review Body and the UK Home Office. Animal care and use adhered to the Animals (Scientific Procedures) Act 1986 and were performed in accordance with a UK Home Office granted Project License.

### Reagents

GM-CSF and M-CSF (Peprotech), IFN-γ and IL-17 ELISAs (R&D), IL-1β, IL-6, IL-10, IL-12p40, and TNF ELISAs and Live/Dead^TM^ fixable dead cell stain kit (Life Technologies) IFN-γ, CD4, IL-17, CD3, DX5, and CD8 flow cytometry antibodies and isotype controls (Biolegend) were used in this study. *Candida albicans* SC5314 was from ATCC. Clinical isolates (*Candida albicans* AM2005/0463, *Candida glabrata* SCS74761 and SCSB5311, *Candida tropicalis* AM2007/0112 and SCS74663, *Candida parapsilosis* AM2005/0207 and SCSB5882) were a gift from Dr. Donna MacCallum (University of Aberdeen).

### Preparation of *Candida* Cultures

*Candida* spp. were plated on YPD agar overnight at 30°C, then cultured in YPD broth for ∼16h at 30°C with shaking, washed three times with PBS by centrifugation and resuspended at the required concentration for experimentation. For some assays, washed *Candida* spp. were killed with 100 mM thimerosal (Merck) for 1h at room temperature. Thimerosal-killed (TK) *Candida* spp. were washed three times with PBS to remove excess thimerosal and resuspended at the required concentration for experimentation.

### *Candida* Cell Wall Isolation and Analysis

*Candida* spp. were grown overnight in YPD broth, washed and broken up with 20 cycles of 45 s at 6 rpm/min using a FastPrep machine (MP Biomedical). The supernatants were centrifuged at 11,000 rpm for 5 min and the pellet contained the cell wall and debris. The pellets were washed with 1 M NaCl and then boiled in 50 mM Tris–HCl buffer pH 7.5, 2% SDS, 0.3 M β-mercaptoethanol and 1 mM EDTA to remove cytoplasmic proteins and membrane proteins. Cell walls were pelleted by centrifugation at 11,000 rpm for 5 min and washed extensively, prior to freeze drying. Acid hydrolysis was then performed on the freeze-dried samples by boiling with trifluoroacetic acid for 3 h. Samples were then prepared at a concentration of 0.1 mg/ml for high pressure liquid chromatography using the CarboPac PA10 column to determine chitin, glucan and mannan levels. 10 μl of prepared samples were injected and run through the column at 1.5 ml/min flow rate with 18 mM sodium hydroxide (NaOH) for 20 min. The column was washed with 200 mM NaOH and equilibrated with 18 mM NaOH prior to the next sample. Standards of monomers; glucosamine (for chitin), glucose (for glucan), and mannose (for mannan) were run under the same conditions.

### Flow Cytometry Analysis of β1,3-Glucan Exposure on *Candida* spp.

5 × 10^6^ live *C. albicans, C. glabrata, C. tropicalis* and *C. parapsilosis*, that had been cultured overnight at 30°C in YPD broth, were incubated with 10 μg of Dectin-1 Fc, Micl Fc or PBS for 2h at 4°C. *Candida* cells were washed with PBS. After washing *Candida* cells were incubated with Pe-conjugated donkey anti-human IgG (Jackson ImmunoResearch) for 30 min. *Candida* cells were washed with PBS, fixed with BD Cytofix (BD Biosciences) and analyzed on an Attune Flow Cytometer (Invitrogen).

### Cell Culture

Bone marrow from the femurs and tibiae of mice were flushed with PBS. Bone marrow derived macrophages (BMDMs) were generated by culturing cells for 6 days in DMEM containing 10% heat inactivated fetal bovine serum, 5% heat inactivated horse serum, 2 mM L-glutamine, 100 U/ml penicillin/streptomycin, 10 mM HEPES and 10ng/ml M-CSF (Patin et al., 2016). BMDCs were generated by culturing cells for 8 days in RPMI 1640 medium containing 10% heat inactivated fetal bovine serum, 2 mM L-glutamine, 100 U/ml penicillin/streptomycin, 10 mM HEPES, 1% NEAA, 1 mM Sodium pyruvate, 50 μM β-mercaptoethanol and 10 ng/ml GM-CSF.

### Cell Stimulations and Cytokine Assays

BMDMs were harvested using 8 mg/ml lidocaine and resuspended in RPMI 1640 containing 10% heat inactivated fetal bovine serum and 100U/ml penicillin/streptomycin. BMDMs and BMDCs were plated at a density of 1 × 10^5^ cells/well of a 96-well plate and left overnight at 37°C. Media was removed, and cells were stimulated with 1 × 10^5^ *Candida* CFUs*/*well for 24 h in a total of 200 μl fresh media. Fungizone (2.5 μg/ml) was added 2h after stimulation. Cell culture supernatants were recovered and assayed for cytokine by ELISA, according to the manufacturer’s protocol.

### *In vivo Candida* spp. Infections

Mice were injected intravenously with 100 μl of *Candida* spp. in PBS. The fungal load inoculated to the mice for specific experiments are outlined in the figure legends. Mice were monitored using a predefined scoring system and weighed daily, with loss of 20% body weight being an additional endpoint. At the end of the experiment, serum, kidneys, brains and spleens were harvested as previously described (Patin et al., 2016). Mice were sacrificed by CO_2_ administration and cervical dislocation. Mice were bled by cardiac puncture and organs were harvested. Serum was centrifuged at 10,000 rpm for 10 min at 4°C in serum tubes and assayed for cytokine by ELISA. The left kidney and right brain were placed in PBS, homogenized and serial dilutions were plated on YPD plates containing 50 μg/ml chloramphenicol, incubated at 30°C for 24–48 h and the colonies were counted per gram organ. The spleen was homogenized and red blood cells were lysed using ACK lysis buffer. Cells were washed and resuspended in IMDM containing 10% heat inactivated fetal bovine serum, 2 mM L-glutamine, 100 U/ml penicillin/streptomycin, 50 μM β-mercaptoethanol. Splenocytes were plated at 1 × 10^6^ cells/well and stimulated with 2 × 10^6^ CFU/wells *Candida* spp. for 48 h at 37°C. Fungizone (2.5 μg/ml) was added 2 h after stimulation. After 48 h, supernatants were recovered and IFN-γ and IL-17 levels were measured by ELISA. Splenocytes were plated at 1 × 10^6^ cells/well and stimulated with PMA/Ionomycin for 4 h at 37°C in the presence of Brefeldin A. IFN-γ and IL-17 producing T-cells were determined by flow cytometry.

### T Cell Flow Cytometry

Following PMA/Ionomycin stimulation, splenocytes were washed with PBS, and stained with live/dead fixable stain (Thermo Fisher Scientific) for 15 min at 4°C. Cells were washed 1× with PBS and 1× with FACS buffer. Cells were blocked with 4 mg/ml 2.4G2 in 5% rabbit serum in FACS buffer for 15 min at 4°C. Cells were stained with CD8, CD4, CD3, NK1.1 antibodies (Biolegend) in FACS buffer for 30 min at 4°C. Cells were washed with FACS buffer, incubated with BD cytofix/cytoperm buffer (BD Biosciences) for 20 min at 4°C. Cells were washed with BD Permwash buffer (BD Biosciences). Cells were blocked with 4 mg/ml 2.4G2 in 5% rabbit serum in FACS buffer for 15 min at 4°C. Cells were stained with IFN-γ and IL17 antibodies for 30 min at 4°C. Cells were washed with BD Permwash and analyzed on a Cyan flow cytometer (Beckman Coulter) or a BD FACS Canto flow cytometer. Data was analyzed using FlowJo software.

### Statistical Methods

Data were analyzed using GraphPad Prism. All data are presented as means ± SEM. Student’s *t* test was used for statistical analysis for two groups or Bonferonni’s post-test after Two-way ANOVA for multiple groups. When data did not follow a Gaussian distribution, it was transformed by *Y* = sqrt(*Y* + 0.5) and analyzed by Student’s *t* test/ANOVA or non-parametric tests if data still did not follow a Gaussian distribution. *p* values less than 0.05 were considered statistically significant: ^*^p < 0.05, ^∗∗^*p* < 0.005, ^∗∗∗^*p* < 0.001.

## Results

### *Candida* spp. Display Differences in Their Cell Wall Composition

As Dectin-1 is important for the immune response to *C. albicans* and *C. glabrata* (Taylor et al., 2007; Marakalala et al., 2013; Chen et al., 2017) we wanted to determine whether it plays a role in response to other medically relevant *Candida* spp. To this end we performed compositional analysis by HPLC of the three major polysaccharides (glucan, mannan, and chitin) in the cell wall of four *Candida* spp. (*albicans*, *glabrata*, *tropicalis*, and *parapsilosis*). The strains of *Candida* used for this analysis were the common *C. albicans* SC5314 lab strain while the *C. glabrata* SCS74761*, C. tropicalis* SCS74663 and *C. parapsilosis* SCSB5882 strains were clinical isolates. Glucan levels were highest in *C. glabrata*, followed by *C. albicans*, *C. tropicalis*, and *C. parapsilosis* (Table 1). The opposite was true for mannan levels as these were highest in *C. parapsilosis* followed by *C. tropicalis*, *C. albicans* and *C. glabrata* (Table 1). Chitin levels were highest in *C. tropicalis*, followed by *C. glabrata, C. albicans*, and *C. parapsilosis* (Table 1). As high levels of glucan are present in the cell wall of each of these different *Candida* spp., Dectin-1 will likely be involved in the immune response to these clinically relevant *Candida* spp.

**TABLE 1:** Relative proportions of carbohydrates in the cell wall extracted from different *Candida* spp.

***Candida* species**	**Chitin**	**Glucan**	**Mannan**
*C. albicans* SC5314	1.72(±0.08)	62.97(±1.1)	35.31(±1.15)
*C. glabrata* SCS74761	2.40(±0.14)	69.51(±0.97)^∗∗∗^	28.09(±1.04)^∗∗∗^
*C. tropicalis* SCS74663	3.24(±0.49)	59.29(±0.42)^∗∗###^	37.47(±0.38)^###^
*C. parapsilosis* SCSB5882	0.37(±0.01)^€^	40.75(±0.3)^∗∗∗###€€€^	58.88(±0.29)^∗∗∗###€€€^

^*^p < 0.05, ^∗∗^*p* < 0.01, ^∗∗∗^*p* < 0.001. ^*^compared to *C. albicans*, ^#^compared to *C. glabrata*, ^€^*compared to C. tropicalis.*

### *Candida* spp. Display Differential β1,3-Glucan Exposure

**FIGURE 1:**
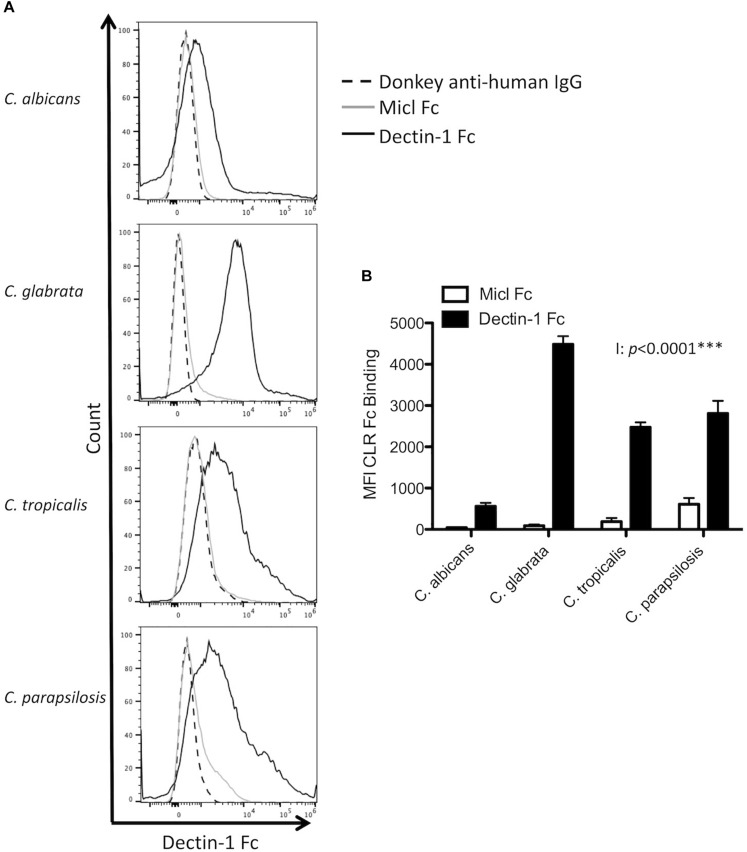
*Candida* spp. display differential β1,3-glucan exposure. **(A,B)** *C. albicans* SC5314*, C. glabrata* SCS74761*, C. tropicalis* SCS74663 and *C. parapsilosis* SCSB5882 were incubated with Dectin-1 Fc, Micl Fc or PBS followed by incubation with Pe-conjugated Donkey anti-human IgG. β1,3-glucan exposure on the *Candida* cell surface was analyzed by flow cytometry. **(A)** Representative flow plots display β1,3-glucan exposure from 3 independent experiments. **(B)** Graphs display MFI of control Micl Fc or Dectin-1 Fc minus MFI of Pe-conjugated Donkey anti-human IgG. Graphs display cumulative data from 3 independent experiments. 2-way ANOVA with Bonferroni’s post-test I = Interaction between *Candida* spp. and CLR Fc binding.

While Dectin-1 binds β1,3-glucan in fungal cell walls (Brown and Gordon, 2001), an important factor in determining Dectin-1 binding is the level of β1,3-glucan exposure (Ballou et al., 2016; Chen et al., 2017). As we observed compositional differences in glucan levels in the four *Candida* spp. (Table 1), we next aimed to determine whether these *Candida* spp. displayed different levels of β1,3-glucan exposure. To this end we stained *Candida* cells cultured in YPD with Dectin-1 Fc or with Micl Fc as a control and quantified mean fluorescence intensity (MFI) by flow cytometry. In agreement with the compositional analysis, *C. glabrata* displayed the highest level of β1,3-glucan exposure (Figures 1A,B). However, *C. albicans* displayed the lowest level of β1,3-glucan exposure, which differs from the compositional analysis, while *C. tropicalis* and *C. parapsilosis* displayed an intermediate level of β1,3-glucan exposure (Figures 1A,B). The four *Candida* spp. tested all have some exposed β1,3-glucan on their cell wall suggesting that Dectin-1 will be involved in the immune response to each of these species, however, differences in the level of β1,3-glucan exposure between the species may control the level of Dectin-1 involvement in various responses.

### Dectin-1 Mediates Cytokine Responses to Multiple *Candida* spp.

**FIGURE 2:**
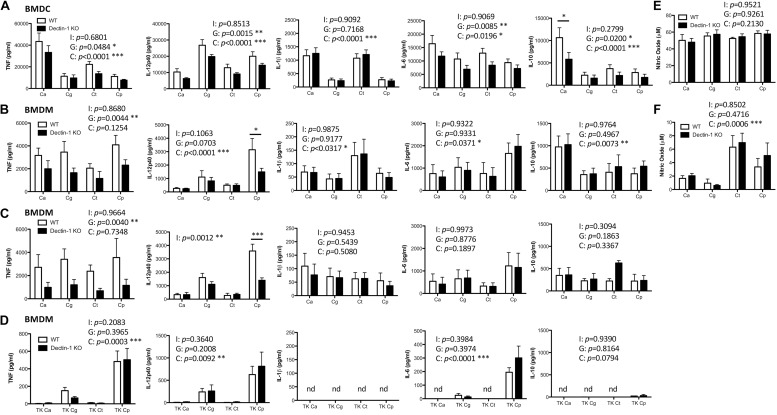
Dectin-1 mediates cytokine responses to multiple *Candida* spp. BMDCs **(A,E)** and BMDMs **(B,F)** from WT and Dectin-1 KO mice were stimulated with *C. albicans* SC5314 (Ca)*, C. glabrata* SCS74761 (Cg)*, C. tropicalis* SCS74663 (Ct) and *C. parapsilosis* SCSB5882 (Cp) at a ratio of 1:1 *Candida*:Cells. **(C)** BMDMs from WT and Dectin-1 KO mice were stimulated with *C. albicans* AM2005/0463*, C. glabrata* SCSB5311*, C. tropicalis* AM2007/0112 and *C. parapsilosis* AM2005/0207 at a ratio of 1:1 *Candida*:Cells. **(D)** BMDMs from WT and Dectin-1 KO mice were stimulated with thimerosal-killed *C. albicans* SC5314 (TK Ca)*, C. glabrata* SCS74761 (TK Cg)*, C. tropicalis* SCS74663 (TK Ct) and *C. parapsilosis* SCSB5882 (TK Cp) at a ratio of 1:1 *Candida*:Cells. Cytokine levels **(A–D)** and nitric oxide levels **(E,F)** in the supernatants were measured after 24h incubation. Results are presented as means ± s.e.m. of 5–6 **(A)**, 4 **(B)**, 3 **(C)**, 2–4 **(D)**, 3 **(E)** or 4 **(F)** independent experiments. (2-way ANOVA, Bonferroni’s post-test) I = Interaction, G = Genotype, C = *Candida*.

Dectin-1 has been shown to mediate *C. albicans-* and *C. glabrata-*induced cytokine production from various myeloid cell populations (Taylor et al., 2007; Chen et al., 2017). Here, we compared the requirement for Dectin-1 to mediate cytokine production in response to four clinically relevant *Candida* spp. To investigate this, BMDCs and BMDMs from WT and Dectin-1 KO mice were stimulated with *C. albicans* SC5314, *C. glabrata* SCS74761*, C. tropicalis* SCS74663 or *C. parapsilosis* SCSB5882. Interestingly, the *Candida* spp. induced rather different cytokine profiles and this varied between BMDCs and BMDMs (Figures 2A,B). We found that TNF production in response to all *Candida* spp. was partially dependent on Dectin-1 in BMDCs and BMDMs (Figures 2A,B). *C. parapsilosis*-induced IL-12p40 production in BMDMs was largely dependent on Dectin-1 (Figure 2B) while it was partially dependent on Dectin-1 in BMDCs in response to all *Candida* spp. (Figure 2A). *Candida*-induced IL-1β production was independent of Dectin-1 (Figures 2A,B). IL-6 production in BMDCs was partially dependent on Dectin-1 while it was independent of Dectin-1 in BMDMs (Figures 2A,B). *C. albicans* induced the highest levels of IL-10 in both BMDMs and BMDCs compared to other *Candida* spp. In BMDCs, IL-10 production was partially dependent on Dectin-1 while it was independent of Dectin-1 in BMDMs (Figures 2A,B). Results were largely similar with an additional strain for each *Candida* spp. (*C. albicans* AM2005/0463, *C. glabrata* SCSB5311*, C. tropicalis* AM2007/0112 or *C. parapsilosis* AM2005/0207) in BMDMs (Figure 2C). In particular, similar to the results in Figure 2B, we found that TNF production in response to all *Candida* spp. was partially dependent on Dectin-1 and *C. parapsilosis*-induced IL-12p40 production in BMDMs was largely dependent on Dectin-1 using additional strains of these 4 *Candida* spp. (Figure 2C). We then killed the *Candida* strains used in Figures 2A,B with thimerosal, which maintains cell wall integrity (Hall et al., 2013), and stimulated WT and Dectin-1 KO BMDMs at an MOI of 1 for 24 h, similar to Figures 2A–C. We found that the thimerosal-killed *Candida* spp. induced much lower cytokine levels (Figure 2D) than live *Candida* (Figure 2B) and in some cases, cytokine production was not detected (nd). Using the thimerosal-killed *Candida* spp., we did not detect significant dependence on Dectin-1 for cytokine production, although as mentioned above cytokine production was substantially lower than that induced by live *Candida* spp. In addition to cytokine production, nitric oxide production is an important immune response to fungal pathogens. We found that *C. tropicalis* and *C. parapsilosis* induced higher levels of nitric oxide in BMDMs compared to *C. albicans* and *C. glabrata*, while all *Candida* spp. induced similar levels of nitric oxide in BMDCs. However, nitric oxide production was independent of Dectin-1 in both BMDMs and BMDCs (Figures 2E,F). Thus, *Candida* spp. induce different cytokine profiles to each other and Dectin-1 mediates *Candida*-induced cytokine responses to live *Candida* spp. but not nitric oxide production, in a cytokine-, cell type-, and *Candida*-dependent manner.

### Dectin-1 Mediates *Candida* Clearance

**FIGURE 3:**
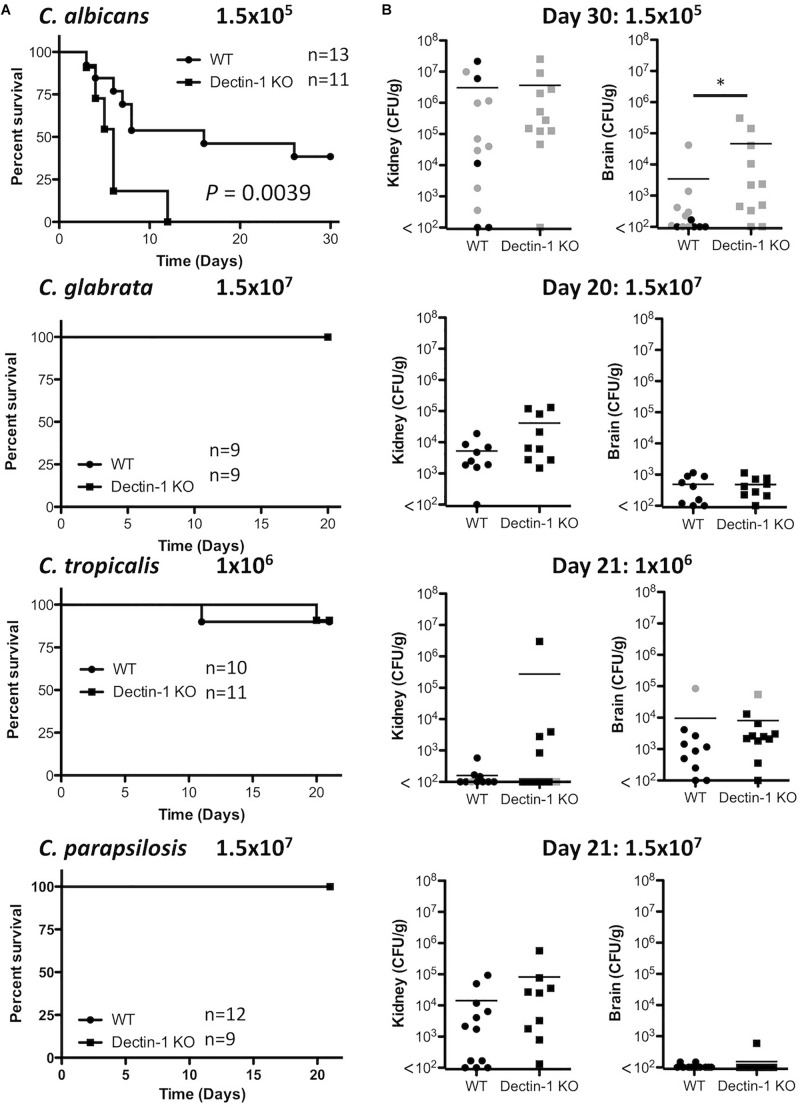
Dectin-1 KO mice are susceptible to infection with highly virulent *C. albicans*. **(A)** WT and Dectin-1 KO mice were infected intravenously with the indicated doses of *C. albicans* for 30 days, *C. glabrata* for 20 days, *C. tropicalis* for 21 days or *C. parapsilosis* for 21 days. Survival curves based on humane end-point of infected WT (filled circles) and Dectin-1 KO mice (filled squares). Graphs are the cumulative result of 2 independent experiments. Log-rank test. **(B)** CFU in the kidneys at 20–30 days after infection (black symbols) or at time of death by humane end point (gray symbols). Graphs are the cumulative result of 2 independent experiments. Each symbol represents an individual mouse. Student’s *t* test on transformed data.

**FIGURE 4:**
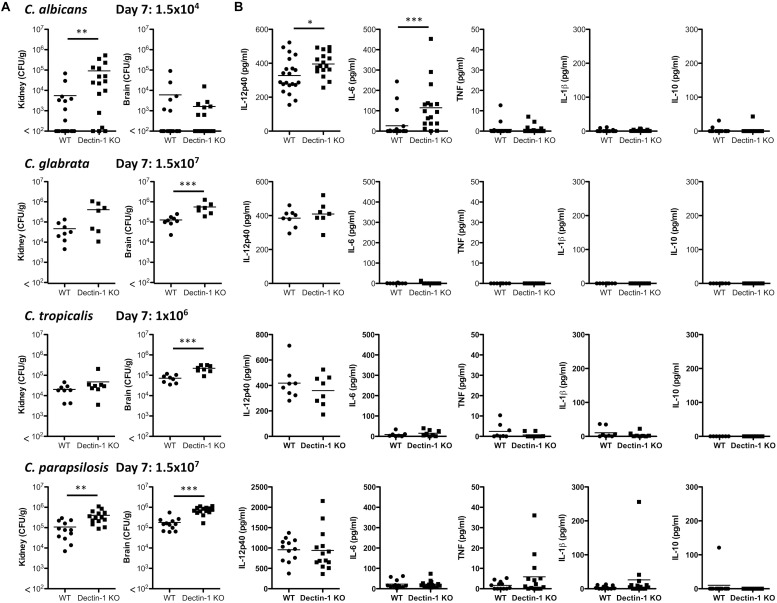
Dectin-1 mediates *Candida* clearance. **(A,B)** WT and Dectin-1 KO mice were infected intravenously with the indicated doses of *C. albicans*, *C. glabrata*, *C. tropicalis* or *C. parapsilosis* for 7 days. **(A)** CFU in the kidneys and brains of mice at 7 days post-infection were determined. Graphs are the cumulative result of 2–4 independent experiments. Each symbol represents an individual mouse. Student’s *t* test (*C. albicans*: transformed data) **(B)** Cytokine levels in the serum of mice at 7 days post-infection. Graphs are the cumulative result of 2–4 independent experiments. Each symbol represents an individual mouse. Student’s *t* test/ Mann-Whitney test (IL-6, TNF, IL-1β, IL-10: transformed data).

As Dectin-1 mediates *Candida*-induced cytokine production (Figure 2), we next compared the role for Dectin-1 in controlling systemic infection with these different *Candida* spp. We first performed an experiment to determine which dose of each *Candida* spp. to use during *in vivo* experiments. Fungal burden in the kidneys of *Candida*-infected mice was compared to mice infected with 1.5 × 10^5^ CFU *C. albicans.* A dose that caused similar kidney fungal burdens was selected for the other strains (Supplementary Figure 1). WT and Dectin-1 KO mice were infected with four *Candida* spp. and in agreement with previous findings (Arendrup et al., 2002), *C. tropicalis*, *C. glabrata*, and *C. parapsilosis* were less virulent than *C. albicans*. Several WT and Dectin-1 KO mice succumbed to infection with *C. albicans*, and Dectin-1 KO mice were more susceptible than WT mice (Figure 3A), similar to previous findings (Taylor et al., 2007). However, mice infected with the other *Candida* spp. (Figure 3A) largely did not succumb to infection even though they were infected with ≈6.6–100× more CFU than *C. albicans*. Dectin-1 KO mice trended toward slightly increased fungal burden in the kidneys and/or brains compared to WT mice at these timepoints (20–30 days) (Figure 3B), although statistically significant increases were not identified at these timepoints for infection with most of the *Candida* spp., We then investigated the importance of Dectin-1 during the early stages of infection. Since many of the *C. albicans*-infected mice succumbed to infection within 1 week (Figure 3A), mice were infected with 10x less *C. albicans* for Figures 4A,B. One week following infection with all four *Candida* spp. Dectin-1 KO mice displayed increased fungal burden in the kidneys and/or brains compared to WT mice (Figure 4A). In addition, *C. albicans*-infected Dectin-1 KO mice displayed increased serum IL-12p40 and IL-6 levels (Figure 4B), likely due to their inability to control the infection. We observed limited TNF, IL-1β, and IL-10 levels in the serum of these mice. Serum cytokine levels in response to other *Candida* spp. were not significantly different in Dectin-1 KO mice (Figure 4B). These data indicate that Dectin-1 is important for surviving infection with the highly virulent *C. albicans*, while it is dispensable for surviving infection with less virulent spp. However, Dectin-1 mediates fungal clearance of multiple *Candida* spp. particularly at early stages of infection.

### Dectin-1 Regulates *Candida*-Associated T Cell Responses

**FIGURE 5:**
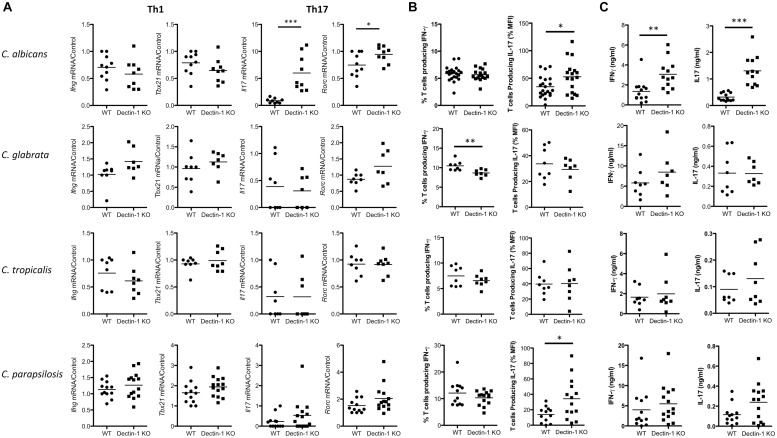
Dectin-1 regulates *Candida*-associated T cell responses. **(A–C)** WT and Dectin-1 KO mice were intravenously infected with 1.5 × 10^4^ CFU *C. albicans*, 1.5 × 10^7^ CFU *C. glabrata*, 1 × 10^6^ CFU *C. tropicalis* or 1.5 × 10^7^ CFU *C. parapsilosis* for 7 days. **(A)** RNA was isolated from the spleens of infected mice, cDNA was prepared and mRNA transcripts were detected by real-time qPCR. mRNA levels were normalized to *Hprt1*. Graphs display the cumulative result of 2 independent experiments. Each symbol represents one mouse. (Student’s *t* test) **(B)** Splenocytes from infected mice were restimulated with PMA/Ionomycin for 4 h and IFN-γ and IL-17 levels were measured by FACS. Graphs display% T cells producing IFN-γ - Isotype or MFI of IL-17 production by T cells. IL-17 MFI by cells from one WT mouse stimulated with PMA/Ionomycin was set at 100% and all other IL-17 MFI in that experiment are presented as a% of this. MFI for the Isotype was then subtracted. **(C)** Splenocytes from infected mice were restimulated with live *Candida* spp. for 48 h and IFN-γ and IL-17 levels were measured by ELISA. **(A–C)** Graphs are the cumulative result of 2–4 independent experiments. Each symbol represents an individual mouse. Student’s *t* test/Mann-Whitney test.

While Dectin-1 is mainly recognized as an important mediator of innate anti-fungal responses, it has also been shown to regulate T cell responses (Robinson et al., 2009; Chen et al., 2017). Therefore, we examined the role of Dectin-1 during T cell responses to four *Candida* spp. 7 days post-infection, splenic *Ifng* and *Tbx21* mRNA levels of all *Candida*-infected mice were similar between WT and Dectin-1 KO mice, however, *Il17* and *Rorc* mRNA levels were increased in *C. albicans*-infected Dectin-1 KO mice compared to WT mice. No major differences were observed in *Il17* levels (low or undetected) or *Rorc* levels in mice infected with the other *Candida* spp. (Figure 5A). We next examined the potential of splenic T cells from these infected mice to produce IFN-γ and IL-17 following PMA/Ionomycin stimulation. This mainly induced a Th1 response with a much smaller Th17 response. IFN-γ producing T cells were somewhat reduced in spleens from *C. glabrata*-infected Dectin-1 KO mice following restimulation with PMA/Ionomycin (Figure 5B and Supplementary Figure 2), while T cells from *C. albicans*- and *C. parapsilosis*-infected Dectin-1 KO mice displayed increased IL-17 production following stimulation with PMA/Ionomycin (Figure 5B). Finally, upon antigen restimulation with live *Candida* spp. in the presence of fungizone, Dectin-1 KO splenocytes displayed increased IL-17 production in response to *C. albicans* (significant) and *C. parapsilosis* (trend) but not *C. glabrata* or *C. tropicalis* (Figure 5C). Similar to our observations with splenic *Il17* mRNA levels, *C. albicans* induced the highest levels of IL-17 upon restimulation compared to other *Candida* spp. Dectin-1 KO splenocytes also displayed enhanced IFN-γ production following restimulation with *C. albicans* but not with other *Candida* spp. These data indicate that Dectin-1 regulates *Candida*-associated T cell responses, in addition to myeloid cell responses.

## Discussion

Here, we have shown that four clinically relevant *Candida* spp. display differences in their polysaccharide composition and β1,3-glucan exposure. We found that *C. glabrata* contained the highest glucan proportion and displayed the most β1,3-glucan exposure, however, we did not find that glucan composition and β1,3-glucan exposure necessarily correlated for the other *Candida* spp. The 4 *Candida* spp. displayed some level of β1,3-glucan exposure and cytokine production in response to all four *Candida* spp. was partially dependent on Dectin-1, however, this varied with *Candida* spp. and cell type. During systemic infections with the various *Candida* spp. we found, similar to others, that *C. albicans* was the most virulent *Candida* spp. out of the four spp. used. Similar to *C. albicans*, we showed that Dectin-1 is important for early clearance of various *Candida* spp. In addition, we found that Dectin-1 regulates some *Candida*-specific T cell responses. Overall, we found that while Dectin-1 mediates/regulates various immune responses to different *Candida* spp., the importance of Dectin-1 for specific immune responses varies for each *Candida* spp.

For this study, we hypothesized that different responses induced by these four clinically relevant *Candida* spp. could potentially be due to the availability of ligands in their cell wall. Analysis of the polysaccharide composition and β1,3-glucan exposure of these four spp. revealed significant differences between the spp. We found that *C. glabrata* displayed the highest β1,3-glucan exposure, *C. tropicalis* and *C. parapsilosis* displayed intermediate exposure and *C. albicans* displayed the lowest level of β1,3-glucan exposure. Apart from *C. glabrata*, there did not appear to be any correlation between glucan level in the cell wall and β1,3-glucan exposure. As the cell wall analysis detects glucan and does not differentiate between β1,3-glucan or β1,6-glucan, this could potentially explain the lack of correlation between the level of glucan in the cell wall and the level of β1,3-glucan exposure or Dectin-1 binding. Regardless of the level of β1,3-glucan exposure, all four spp. displayed some dependence on Dectin-1 for various immune responses, however, we did not observe any clear correlation of the level of glucan in the cell wall or the level of β1,3-glucan exposure with dependence on Dectin-1. Further analysis of multiple strains of each *Candida* species would be required to determine whether the differences in cell wall composition or ligand availability are reflective of the species or of a particular strain. In fact, various groups have previously examined polysaccharide composition and β1,3-glucan exposure on some of these *Candida* spp. and these results sometimes differ from ours and from each other (Linden et al., 2010; Estrada-Mata et al., 2015; Sem et al., 2016). For example, one group showed that *C. parapsilosis* contains a higher % glucan content that *C. albicans*, which differs from our results (Estrada-Mata et al., 2015). However, another group showed that *C. parapsilosis* displays more β1,3-glucan exposure than *C. albicans*, which is in agreement with our results (Linden et al., 2010). In addition, another group showed that β1,3-glucan exposure increased from *C. tropicalis*, *C. albicans*, *C. glabrata* to *C. parapsilosis* (Sem et al., 2016). These data suggest that glucan composition and β1,3-glucan exposure vary from one *Candida* strain to another and these differences are not necessarily reflective of *Candida* spp. rather each individual strain. Differences in experimental conditions and *Candida* strains between research groups could potentially explain these different results. We and others have examined β1,3-glucan exposure at a particular point, usually after overnight culture in YPD broth. These *Candida* spp. differ in their ability to form yeast, pseudohyphae or hyphae (Gow et al., 2011; Silva et al., 2012), and in an *in vitro* assay and *in vivo* these *Candida* spp. will undergo changes that will modify the level of β1,3-glucan exposure over time. In fact, Ballou et al. (2016) showed that lactate in the growth media masks β1,3-glucan compared to glucose in the growth media. In addition, β1,3-glucan, chitin and/or mannan exposure can be affected by the addition of neutrophils and/or normoxic versus anoxic culture conditions (Lopes et al., 2018). Furthermore, Marakalala et al. (2013) showed that Dectin-1 dependence *in vivo* was determined by changes in the fungal cell wall chitin content that occurred *in vivo*. Therefore, many factors will influence β1,3-glucan exposure and Dectin-1 dependence over time for various immune responses, so it is not surprising that we did not observe direct correlations between the level of β1,3-glucan exposure at one particular timepoint with the level of Dectin-1 involvement during various anti-fungal responses.

In this study, we assessed the involvement of Dectin-1 in mediating *Candida*-induced cytokine production from BMDCs and BMDMs. In general, we found that the *Candida* spp. induced much higher levels of cytokines from BMDCs than from BMDMs. Similar to previous findings with *C. albicans* (Gow et al., 2007; Taylor et al., 2007; Chen et al., 2017), we found that Dectin-1 partially mediates TNF production to the various *Candida* spp. in both BMDCs and BMDMs. We also found that Dectin-1 partially mediates IL-12p40 production from BMDCs in response to various *Candida* spp. and it largely mediates IL-12p40 production in response to *C. parapsilosis* from BMDMs. In addition, Dectin-1 somewhat contributes to *C. glabrata*-induced IL-12p40 production by BMDMs similar to previous findings (Chen et al., 2017). IL-6 and IL-10 production in response to the various *Candida* spp. was partially dependent on Dectin-1 in BMDCs but not BMDMs. Various groups have shown that Dectin-1 mediates cytokine production such as TNF, IL-6, IL-12p20 and IL-10 from monocytes, macrophages or DCs to *Candida* spp., zymosan or curdlan (Gow et al., 2007; LeibundGut-Landmann et al., 2007; Taylor et al., 2007; Estrada-Mata et al., 2015; Chen et al., 2017), whereas others have shown that Dectin-1 is dispensable for these responses in macrophages (Saijo et al., 2007) or BMDCs (Robinson et al., 2009). Several factors could contribute to the different results observed including the source of the macrophages (peritoneal, alveolar, BMDM), cell culture conditions, type of ligand used (live/killed *Candida* spp., zymosan, curdlan), background of the mice (C56BL6, 129/Sv) and human or mouse cells. In addition, as discussed above, β1,3-glucan exposure and whether chitin or mannans are masking the β1,3-glucans could all help to determine whether Dectin-1 is involved in any particular experimental setup. Furthermore, the role of additional receptors such as Dectin-2, Mannose Receptor, TLR2 and TLR4 likely differs due to receptor expression on different cell types, exposure of the relevant ligands on different *Candida* spp. and interaction with the other *Candida*-interacting receptors on each particular cell type (Netea et al., 2006; Hall et al., 2013; Ifrim et al., 2014, 2016; Estrada-Mata et al., 2015; Perez-Garcia et al., 2016). When the *Candida* spp. were killed with thimerosal, we observed much lower levels of cytokine production from BMDMs than with live *Candida* spp. and we did not observe any significant Dectin-1 dependence. While thimerosal maintains cell wall integrity (Hall et al., 2013), the *Candida* cells are then unable to undergo any changes due to different growth media, encountering host immune cells or differences in oxygen levels that may alter β1,3-glucan exposure or masking. Various studies have previously found increased responses to heat killed *C. albicans* and major dependence on Dectin-1 in response to heat killed *C. albicans*, compared to live or thimerosal killed *C. albicans*, as this exposes β1,3-glucans (Gow et al., 2007; Hall et al., 2013; Estrada-Mata et al., 2015). Without increased exposure of β1,3-glucans either due to heat killing or due to different culture conditions, the thimerosal killed *Candida* spp. may not be able to induce major Dectin-1 dependent responses. Overall, we found that different cytokine profiles are produced dependent on cell type, *Candida* spp. and/or strain, and *Candida* cell viability.

In addition to mediating innate myeloid cell cytokine responses, we found that in some cases effector T cell responses are enhanced in cells from Dectin-1 KO mice. Previous studies have shown that following antigen restimulation splenocytes from Dectin-1 KO mice displayed reduced IFN-γ and/or IL-17 production, however, these studies used heat-killed or UV-inactivated *Candida* spp. (Robinson et al., 2009; Chen et al., 2017). As heat-killing and UV-inactivation alters the fungal cell wall and exposes different ligands thereby increasing the dependence on Dectin-1 (Gow et al., 2007; Estrada-Mata et al., 2015), this could potentially explain the difference in our results. Our data demonstrate an enhanced generic and antigen-specific Th17 response (*albicans* and *parapsilosis*) and an enhanced antigen-specific IFN-γ response (*albicans*) in Dectin-1 KO mice, likely due to increased exposure of T cells to *Candida in vivo* due to the inability to control these infections. However, the fungal burden is increased in Dectin-1 KO mice during infection with each of the *Candida* spp. while IL-17 responses are not increased in response to *C. glabrata* or *C. tropicalis*. Therefore, in addition to increased fungal burden it is possible that Th17 promoting cytokines such as TGF-β, IL-6 and IL-23 are differentially regulated *in vivo* in response to the various *Candida* spp. (Sandquist and Kolls, 2018). In support of this theory, serum levels of IL-6 are increased in Dectin-1 KO mice during infection with *C. albicans*, and cells from these mice demonstrate the highest *Candida*-induced IL-17 response. It is also possible that the enhanced Th17 effector response may at least in part be mediated by Dectin-2 as Dectin-2 has been shown to induce Th17 responses to *C. albicans* (Saijo et al., 2010). The enhanced Th17 response in Dectin-1 KO cells could potentially be a compensatory mechanism by Dectin-2 to deal with the lack of a Dectin-1 response. Thus Dectin-1 is important for both innate and adaptive immune responses to multiple *Candida* spp. although it’s role in specific responses varies with different *Candida* spp.

## Conclusion

The present study shows that multiple clinically relevant *Candida* spp. induce significantly different innate cytokine profiles and that Dectin-1 mediates these cytokine responses in a cell type and *Candida* spp. dependent manner, indicating that results with *C. albicans* cannot necessarily be fully extrapolated to other *Candida* spp.

## Data Availability

All datasets generated for this study are included in the manuscript and/or the Supplementary Files.

## Author Contributions

AT designed and performed the experiments and wrote the manuscript. JG, LW, DF, and KL performed the experiments. PT and NG guided the research. SO conceptualized and guided the research, designed and performed the experiments, and wrote the manuscript. All authors contributed to the manuscript revision, and read and approved the submitted version.

## Conflict of Interest Statement

The authors declare that the research was conducted in the absence of any commercial or financial relationships that could be construed as a potential conflict of interest.
